# Postnatal DNA demethylation and its role in tissue maturation

**DOI:** 10.1038/s41467-018-04456-6

**Published:** 2018-05-23

**Authors:** Yitzhak Reizel, Ofra Sabag, Yael Skversky, Adam Spiro, Benjamin Steinberg, Diana Bernstein, Amber Wang, Julia Kieckhaefer, Catherine Li, Eli Pikarsky, Rena Levin-Klein, Alon Goren, Klaus Rajewsky, Klaus H. Kaestner, Howard Cedar

**Affiliations:** 1 Department of Developmental Biology and Cancer Research, Hebrew University Medical School, P.O.B. 12272, , Jerusalem, 91120 Israel; 2 Department of Genetics and Institute for Diabetes Obesity and Metabolism, Perelman School of Medicine, University of Pennsylvania, 12-126 Translational Research Center, 3400 Civic Center Boulevard Building 421, Philadelphia, PA 19104-5156 USA; 3 Broad Technology Labs (BTL), The Broad Institute of Harvard and MIT, Cambridge, MA 02142 USA; 4 Department of Biochemistry, Molecular Biology and Biophysics, University of Minnesota, Minneapolis, MN 55455 USA; 5 Department of Medicine, University of California San Diego, La Jolla, CA 92093 USA; 6 Immune Regulation and Cancer Max-Delbrück-Centrum für, Molekulare Medizin (MDC), Robert-Rössle-Strasse 10, Berlin 13092 Germany

## Abstract

Development in mammals is accompanied by specific de novo and demethylation events that are thought to stabilize differentiated cell phenotypes. We demonstrate that a large percentage of the tissue-specific methylation pattern is generated postnatally. Demethylation in the liver is observed in thousands of enhancer-like sequences associated with genes that undergo activation during the first few weeks of life. Using a conditional gene ablation strategy we show that the removal of these methyl groups is stable and necessary for assuring proper hepatocyte gene expression and function through its effect on chromatin accessibility. These postnatal changes in methylation come about through exposure to hormone signaling. These results define the molecular rules of 5-methyl-cytosine regulation as an epigenetic mechanism underlying cellular responses to a changing environment.

## Abstract (web summary)

Here the authors show that a large fraction of the tissue-specific methylation pattern is generated postnatally. These changes, which occur in response to hormone signaling, appear to play a major role in the regulation of gene expression and tissue maturation in the liver.

## Introduction

DNA methylation patterns are established during mammalian embryogenesis through a programmed process that involves both de novo and demethylation events. Initially, most methyl groups are erased in the early embryo before a new bimodal pattern is established at the time of implantation, where almost all CpG sites undergo de novo methylation while CpG islands are protected by virtue of *cis*-acting regulatory sequences^1^. Following implantation, all subsequent changes in DNA methylation appear to be gene specific, with pluripotency genes, for example, becoming de novo methylated at the time of gastrulation^2,3^, while tissue-specific regulatory regions undergo demethylation during embryonic development^4,5^ or during adult stem cell differentiation^6^. These processes are triggered by external signaling factors, which then direct interactions between *cis*-acting sequences and *trans*-acting proteins within the cell that have the ability to recruit either the de novo methylation complex or demethylation machinery^1^. Once the epigenetic alterations occur, they serve to autonomously maintain these developmental decisions by generating a stable chromatin template that can persist for long time periods^1^.

It is generally believed that the DNA methylation patterns established during development do not undergo further alterations, but recent studies indicate that methylation changes may indeed occur postnatally in terminally differentiated tissues. This has been observed, for example, in cardiomyocytes where many gene regions become hypomethylated or hypermethylated during heart muscle maturation^7^ and stable programmed demethylation of defined regulatory sites also occurs postnatally in the mammary tissue following pregnancy^8^ as well as in the liver^9,10^, where some of these events are mediated by the secretion of testosterone^11^. It has been suggested that DNA methylation changes may also be induced by the environment or by specific behaviors^12^. In this paper, we have used high-throughput analysis to demonstrate the existence of programmed postnatal changes in methylation in many different tissues of the body. Furthermore, using the liver as a model system, we show that these changes, which are induced by hormone signaling, are associated with extensive alterations in gene expression that take place during the first few weeks after birth. Finally, by employing genetic intervention, we show that these methylation changes play a role in the process.

## Results

### Postnatal demethylation

**Fig. 1:**
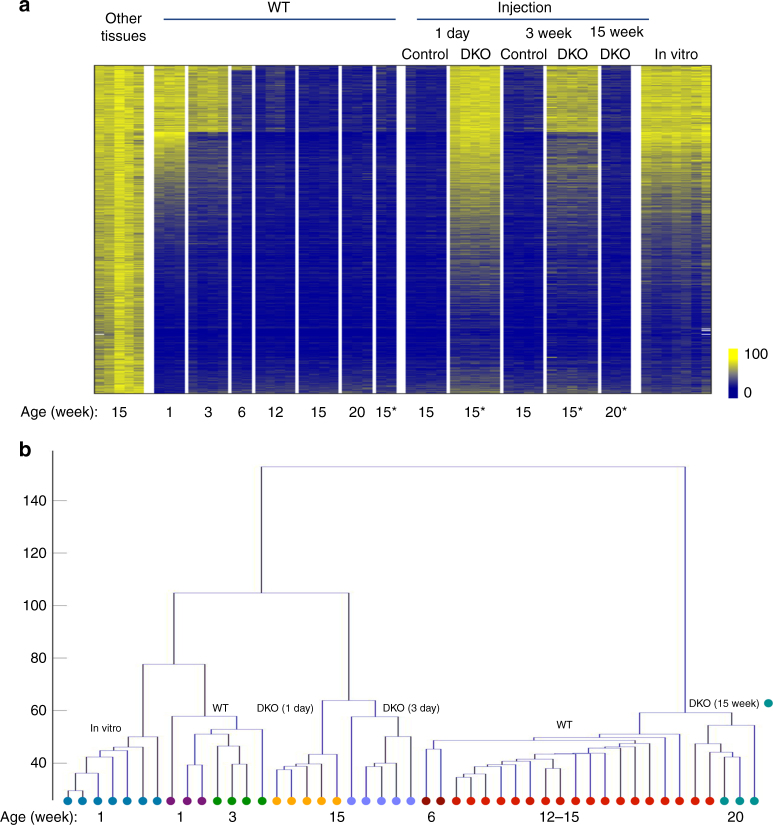
Postnatal demethylation in the liver. **a** Heatmap of specifically undermethylated 100 bp regions (*n* = 9000) measured in hepatocytes from WT or Tet2^F^/Tet3^F^ (*) animals at different ages and in other tissues (lung, heart, neutrophils, brain, fat) by RRBS. Animals were injected with AAV8-expressing Cre under the Tbg-Cre promoter at 1 day, 3 weeks, or 15 weeks (control or DKO). Note that there is only a slight degree of remethylation (<6%) in the 3-week DKO samples. Hepatocytes were also isolated from newborn mice and cultured in vitro for 3 weeks. **b** Unbiased hierarchical clustering analysis on the entire hepatocyte RRBS data set without selection for specific sequences, including the injection controls and in vitro grown hepatocytes (1 week). Injected control samples are included in the 12–15 weeks group

In order to investigate the possibility that methylation patterns are subject to postnatal alterations, we analyzed genome-wide methylation in the mouse liver from the time of birth through adult life. This was accomplished using reduced representation bisulfite sequencing (RRBS), a technique that covers a large fraction of the regulatory sequences present in DNA and at sufficient depth to obtain reproducible results^13^. By choosing strict criteria to characterize DNA methylation changes (see Methods), we found over 9000 tiles (100 bp) that are undermethylated (>50% reduction) specifically in the adult liver as compared to DNA from a panel of other cell types (*p* value <10^−4^, permutation test). While most of these sites are already unmethylated in 5-day-old liver (1 week), about 2800 actually undergo significant demethylation (>35%) postnatally, with a large number of these (~1800) losing their methyl groups only after 3 weeks (Fig. 1a). Unbiased clustering analysis of the entire RRBS data set showed that these differentially methylated regions (DMRs) are highly reproducible and statistically robust (Fig. 1b). Interestingly, even though many regions undergo specific de novo methylation in the liver, only a small number of these sites (~10%) become modified postnatally (Supplementary Fig. 1a). Of note, all of these measurements were carried out on purified hepatocytes, thus verifying that this process is truly cell autonomous and not the result of changes in cell composition during maturation of the liver.

### Biochemical mechanism of demethylation

Demethylation during early stages of embryogenesis is mediated by combinations of TET enzymes which hydroxylate 5mC^14–19^, thus setting the stage for replacement either through replication dilution or active DNA repair processes^20–23^. This also seems to be the case for the postnatal demethylation observed in the liver, as indicated by whole-genome analysis (see Methods), demonstrating that these DMRs are highly enriched for 5hmC (Supplementary Fig. 2). Recently, it was demonstrated that the specific combination of TET2 and TET3 is necessary for the demethylation that takes place during B cell development in vivo^24^. In order to test whether postnatal demethylation in the liver is also accomplished in this manner, we first verified that both *Tet2* and *Tet3*, but not *Tet1*, are expressed in hepatocytes. Tet2^F^/Tet3^F^ mice^25,26^ were then injected either at 1 day or 3 weeks after birth with adeno-associated virus 8 (AAV8) carrying the gene for Cre recombinase under the control of the hepatocyte-specific thyroid-binding globulin (Tbg) promoter^27^ and followed until they reached the age of 15 weeks. Preliminary control experiments indicated that infection with AAV in this manner works efficiently to delete target sequences (Supplementary Fig. 3).

Using both RRBS (Fig. 1) and whole-genome bisulfite sequencing (WGBS) (Supplementary Fig. 2), we then analyzed all the sites that normally undergo demethylation in the liver. Double mutants (DKO) of Tet2/Tet3 at birth completely prevented subsequent demethylation. Notably, while some of the regions begin to undergo demethylation immediately after birth, almost half of them are demethylated much later, after the first three weeks of life. Removal of *Tet2* and *Tet3* by infection with AAV8-cre at 3 weeks was able to inhibit demethylation at these sites, but regions that had already undergone demethylation prior to AAV8-cre administration remained largely hypomethylated in these animals (<6% increase). Similar results were obtained when gene ablation was carried out in older animals (15 weeks) following completion of all demethylation events (Fig. 1). This is consistent with the notion that once established, the unmethylated state is autonomously maintained without the need for continuous demethylase activity^28^. Taken together, these results show that postnatal demethylation in the liver is mediated by TET2/TET3 and that in the absence of these enzymes the methylation pattern in hepatocytes remains invariant (Fig. 1). It should be noted that the double mutant had no effect on postnatal de novo methylation (Supplementary Fig. 1a).

**Fig. 2:**
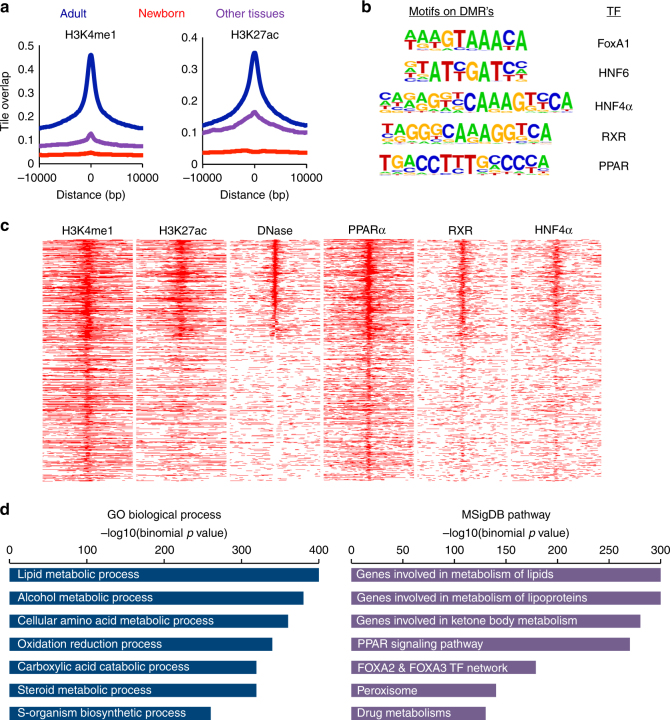
Characterization of regions that undergo postnatal demethylation in hepatocytes. **a** ChIP-Seq of demethylated regions (52,000 200 bp tiles as determined by WGBS) as a function of distance from their center for H3K4me1 and H3K27ac in adult liver (blue), newborn liver (red), and other tissues (purple). **b** Transcription factor motif analysis of these same demethylated regions (*p* value <10^−300^ for all motifs). **c** Heatmaps of H3K4me1 and H3K27ac, DNaseI, PPARα, and HNF4α ChIP binding in hepatocytes as a function of distance from the DMR center (±10 kb) ranked according to the DNaseI lane. **d** Gene ontology and pathways associated with postnatal demethylated regions

To gain insight into the nature of DNA segments that undergo postnatal demethylation in the liver, it was necessary to generate a more comprehensive picture of this phenomenon, and to this end, we carried out WGBS analysis on a limited number of hepatocyte samples. This experiment, which was done at a sequencing depth (>30-fold) that allowed the accumulation of highly significant data, revealed approximately 52,000 regions that undergo demethylation postnatally (Supplementary Dataset 1 and Supplementary Fig. 2) with an average size of about 250 bp (Supplementary Fig. 2c). These regions are distributed within a distinct window around its nearest transcription state site (TSS), including both intragenic and intergenic regions of the genome, with very few (<2%) located in promoter regions themselves (Supplementary Fig. 2d, e). Analysis of published chromatin immunoprecipitation-sequencing (ChIP-Seq) data indicated that these regions are highly enriched for histone H3K4me1 and H3K27ac in the adult liver, but not in other tissues or liver from newborn animals (Fig. 2a).

### Enhancer demethylation mediates gene expression

This finding suggests that these sequences may represent enhancer elements that become activated postnatally and bioinformatics analysis shows that they are indeed enriched for a number of different common enhancer-binding motifs (genomatix genome analyzer, https://mygga.genomatix.de/) of transcription factor families known to be important in hepatocytes (Fig. 2b). Indeed, many of these sites have a DNaseI-sensitive conformation indicative of protein binding, and several of these factors bind to their demethylated target sequences in the adult liver (Fig. 2c). This suggests that postnatal demethylation occurs at regulatory sites and Gene Ontology (GO) analysis indicates that these sequences are located near genes that are highly enriched for various aspects of liver function (Fig. 2d). Indeed, over 4500 of these DMRs actually represent elements within previously identified hepatocyte super-enhancers^29^.

**Fig. 3:**
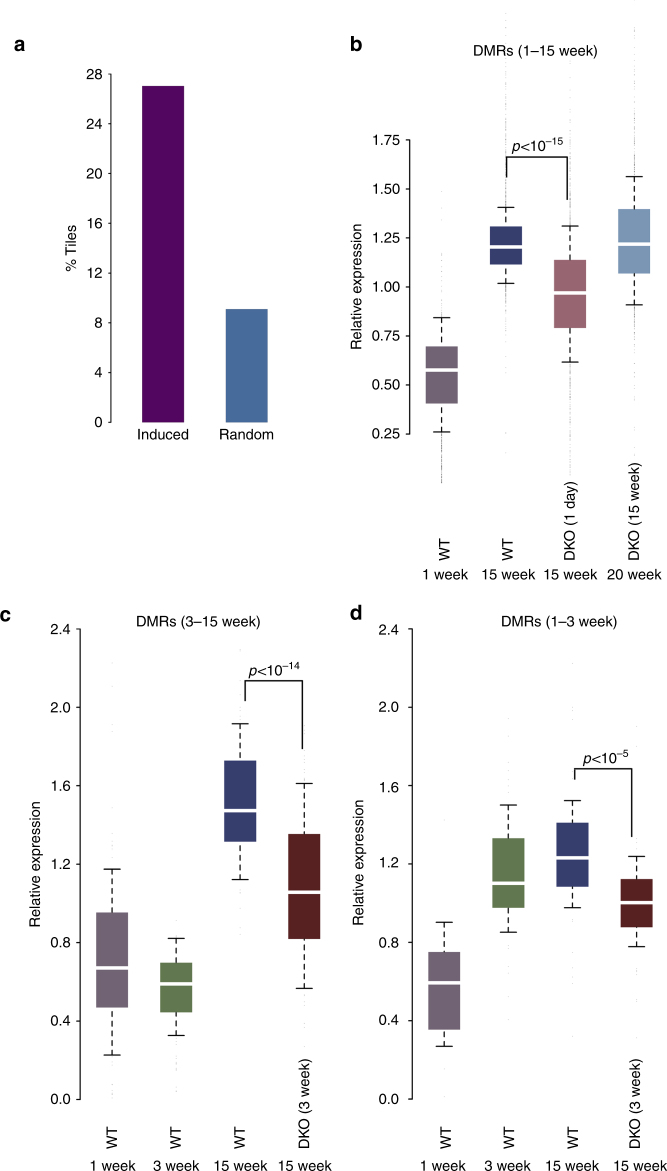
Demethylation and gene expression. **a** Percentage of DMRs (WGBS) located in genes (see Methods) that are upregulated with age (*n* = 8500) compared to randomly selected DNA segments (*p* value <10^−27^, *z*-test of proportions). **b** Box plots of relative expression (normalized fragments per kilobase million (FPKM)) in WT and Tet2/Tet3-deficient hepatocytes for 1212 genes upregulated after birth that contain intragenic DMRs. The DKO was induced at either 1 day or 15 weeks. Similar results were obtained using WT animals injected with AAV8-cre. **c** Expression of 115 genes that undergo demethylation (RRBS) and that are upregulated between 3 and 15 weeks. The DKO was induced at 3 weeks. **d** Expression of 47 genes that undergo demethylation (RRBS) and that are upregulated between 1 and 3 weeks. The DKO was induced at 3 weeks. In the DKO, the pattern of expression at 15 weeks was very similar to that of WT animals at 3 weeks

In order to test whether changes in methylation that occur postnatally may also have an effect on gene expression, we carried out RNA-Seq analysis on hepatocyte samples from newborn, 3-week-old and 15-week-old animals. Under the assumption that the DMRs detected in the liver are enhancer-like regulatory sequences, we first analyzed the ~31,000 sites that are located within or adjacent to known genes (see Methods and Supplementary Fig. 4). About 30% of these regions (*n* = 8500) are associated with genes (*n* = 1212) that show a significant (*p* value <10^−27^, *z*-score of proportions) postnatal increase in mRNA levels (Fig. 3a). This represents over 60% of all genes (*n* = 2070) that are upregulated during liver maturation between 1 and 15 weeks. Moreover, this increase in transcription was significantly inhibited in Tet2/Tet3 double mutant animals (Fig. 3b and Supplementary Fig. 5).

As a control, we examined mice that underwent gene ablation at 15 weeks, well after completion of all demethylation events and found that removal of *Tet2/Tet3* had almost no effect on the transcription of these genes. This clearly indicates that it is demethylation at these enhancer sequences itself that plays a direct role in gene induction. We next examined the effects of deleting *Tet2/Tet3* at 3 weeks. As expected, DMRs that undergo demethylation after this point are associated with genes whose expression begins to increase after 3 weeks, and these are substantially affected by loss of the TET enzymes (Fig. 3c). In contrast, the expression of genes containing DMRs that have already undergone demethylation prior to 3 weeks was largely unaffected by the late gene deletion (Fig. 3d), again supporting the idea that the main effect of TET2/TET3 is at the level of demethylation.

It should be noted that about 70% of the intragenic DMRs (*n* = 22,659) are associated with genes that do not undergo significant postnatal induction (Supplementary Fig. 4). Nonetheless, these sites, as well as the intergenic DMRs (*n* = 21,023), appear to be packaged into nucleosomes containing the enhancer marks H3K4me1 and H3K27ac, are sensitive to DNaseI, and bind liver-specific transcription factors to the same extent as those located within the genes that do undergo postnatal transcriptional activation (Fig. 2 and Supplementary Fig. 4), suggesting that these DMRs may also be involved in gene activation. In order to test this possibility, we took advantage of chromosome conformation capture (Hi-C)^30^ data obtained from liver cells, and in this way, physically identified additional interactions between DMRs and postnatally induced genes. Taken together, these data indicate that the DMRs identified in this study interact with and may influence a relatively large percentage of all postnatally induced genes.

### Functional mechanism of demethylation

**Fig. 4:**
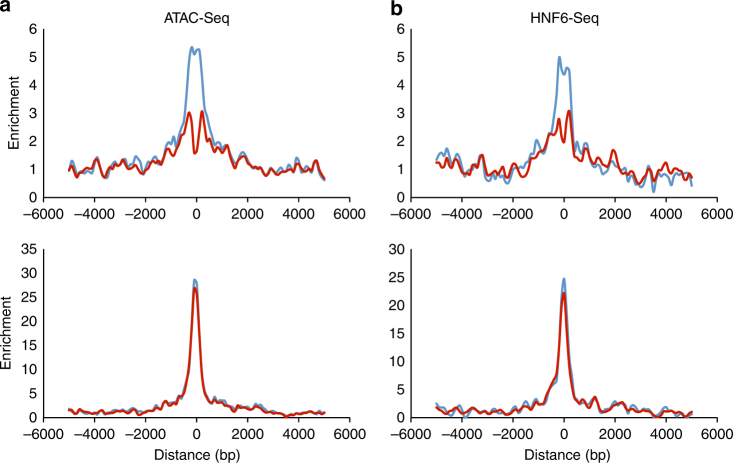
Effect of DNA methylation on chromatin accessibility. **a** ATAC-Seq of postnatal DMRs (2800 100 bp regions as determined by RRBS) as a function of distance from their center in WT (blue) and DKO (red) adult (15 weeks) hepatocytes. Also shown for comparison (lower panel) is an analysis of hepatocyte-specific prenatal DMRs that are already fully unmethylated (<5%) in 1 week hepatocytes (*n* = 1290). **b** ChIP-Seq of postnatal and prenatal DMRs for HNF6 as a function of distance from their center tile. All curves represent the average of two independent experiments

Since demethylation appears to be important for achieving proper levels of gene expression, we next took advantage of our Tet2/Tet3-deficient mice to ask whether this mechanism operates by affecting gene structure. To this end, we carried out an assay for transposase-accessible chromatin with high-throughput sequencing (ATAC-Seq) on both wild-type (WT) and Tet2/Tet3-deficient (1 day) adult hepatocytes and specifically examined the postnatal DMR regions. Strikingly, while many DMRs were detected in this assay, these same exact sites were found to be locally inaccessible in the mutants (Fig. 4a and Supplementary Fig. 6). This is in contrast to prenatally formed DMRs that are unaffected by Tet2/Tet3 deficiency and therefore serve as a negative control. In addition, ChIP-Seq analysis shows that binding of the liver-enriched transcription factor HNF6 is also reduced in the presence of DNA methylation (Fig. 4b and Supplementary Fig. 6). Although it has been previously demonstrated that DNA methylation affects chromatin structure^31,32^, our experiments represent the first definitive proof of this principle in vivo. It should be noted that DNA methylation only had a very minor influence on the presence of histone markers H3K4me1 and H3K27ac, as shown by ChIP-Seq analysis, probably because these modifications tend to have a regional pattern that may not be significantly affected by local changes in DNA methylation (Supplementary Fig. 7).

### Phenotype of Tet DKO

**Fig. 5:**
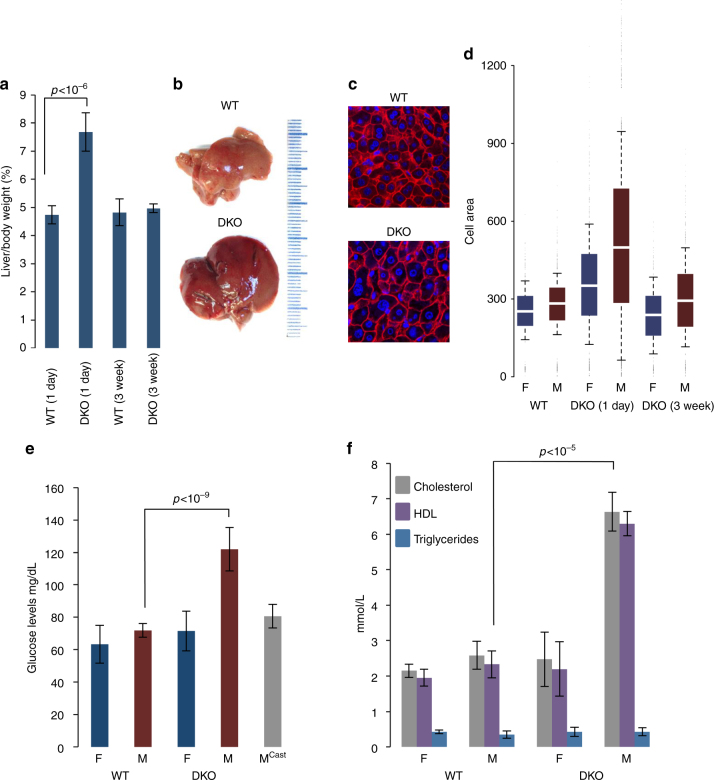
Effect of Tet2/Tet3 deficiency on liver function. **a** Liver to body weight ratio (±s.d.) in 15-week-old WT and DKO mice induced at 1 day or 3 weeks. **b** WT and DKO (1 day) liver. **c** E-cadherin staining of sections from 15-week-old WT and DKO mice induced at 1 week. **d** Box plot of hepatocyte cell size (μ^2^) based on E-cadherin staining in 15-week-old WT and DKO male (M) and female (F) mice induced at 1 day or 3 weeks. **e** Serum glucose or **f** cholesterol, HDL, and triglyceride levels (±s.d.) of 15-week-old WT and DKO mice. Results are also shown for DKO males castrated at 3 weeks (M^Cast^)

Considering the extensive changes in methylation and transcription that are seen in the Tet2/Tet3 double mutant, we next asked whether these molecular indicators are also reflected in liver function (Fig. 5). In all of the 1 day mutant mice, both males and females, we observed an increased liver mass to body weight ratio of about 60% compared to control mice (Fig. 5a, b) and histological analysis indicated that this may be due to increased cell size as measured quantitatively by E-cadherin staining (Fig. 5c, d). We also carried out blood biochemistry in order to determine whether the lack of demethylation brings about metabolic changes. This revealed that while most biochemical indicators of liver function are not altered in the Tet2/Tet3-deficient animals, there were significant increases in blood glucose, cholesterol, and high-density lipoprotein (HDL) in all male mutant mice, but not in females, whether the gene ablation was induced early (1 week) or late (3 weeks). This suggests that the effect on metabolism may come about because of a lack of male-specific testosterone-mediated late demethylation (after 3 weeks)^11^, as confirmed by showing that these biochemical changes do not occur in castrated mice (Fig. 5e). In keeping with this, bioinformatic analysis indicated that a number of genes associated with male-specific DMRs are indeed functionally involved in glucose (e.g., *Fto*, *Insr*, *Gpd2*, *Gsk3b*) and lipid (e.g., *Lpin1*, *Scp*2, *Cyp7b*1, *Fabp6*) metabolism possibly explaining the DKO phenotype (Supplementary Fig. 8).

### Demethylation is mediated by insulin signaling

**Fig. 6:**
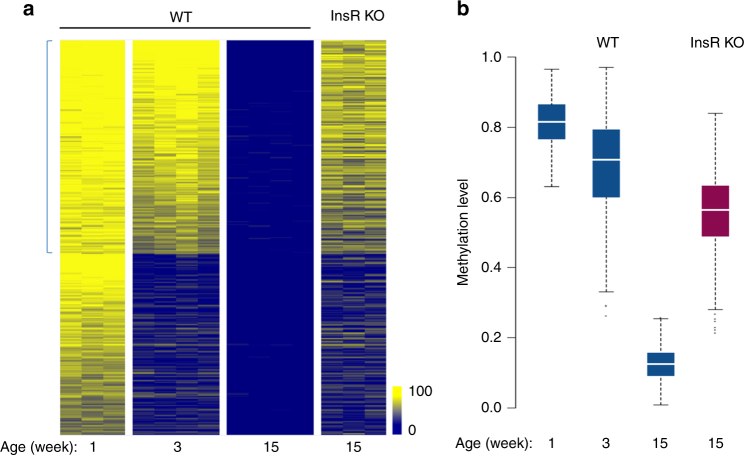
Effect of insulin receptor ablation on postnatal demethylation. **a** Methylation heatmap (RRBS) showing 2800 DMRs from WT and InsR-deficient (1 day) hepatocytes that undergo demethylation postnatally from 1 week to 15 weeks. Note that InsR ablation affects mainly those sites (*n* = 1520) that undergo demethylation after 3 weeks (marked with blue bar). **b** Box plot of methylation levels for these 1520 DMRs

Initial studies indicated that newborn hepatocytes cultured for several weeks in vitro still maintain the same methylation pattern as that seen in 1-week-old animals (Fig. 1). This suggested that the postnatal alterations we observed are not an intrinsic part of the hepatocyte methylation program, but rather depend on factors found in vivo that are evidently not present in the culture medium. Indeed, many different factors and hormones are known to influence neonatal development, with insulin playing a central role in this process^33^. To test whether this hormone may play a role in hepatic postnatal demethylation, we took advantage of the existing InsR^F/F^ mouse^34^ and injected it right after birth with AAV8 Tbg-Cre. These animals, which are defective for the InsR specifically in hepatocytes, were analyzed by RRBS and compared to WT animals at 15–20 weeks. Strikingly, about 40% of the normal DMRs remained highly methylated in the mutant hepatocytes (Fig. 6), clearly indicating that insulin signaling is necessary, directly or indirectly, for a major portion of the methylation changes that occur postnatally. It should be noted that the InsR ablation affected almost exclusively DMRs that are formed after 3 weeks. This is consistent with the observation that other insulin-signaling-dependent effects occur at this stage^35^, perhaps because of the change in insulin levels that take place during the first two weeks of life^36^.

### Postnatal demethylation in other tissues

**Fig. 7:**
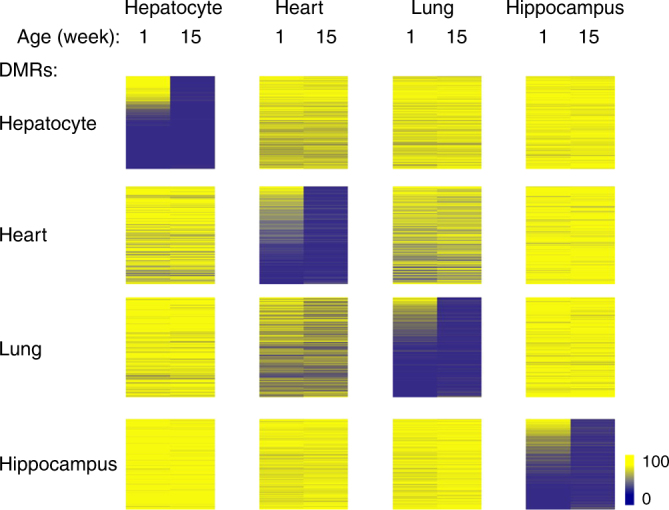
Postnatal demethylation in various tissues. Methylation heatmap (RRBS) of hepatocyte-specific (*n* = 9408), heart-specific (*n* = 892), lung-specific (*n* = 914), and hippocampus-specific (*n* = 3630) DMRs (*p* value <10^−4^, permutation test) ranked according to newborn methylation levels in each tissue

We next asked whether postnatal demethylation represents a general phenomenon. To this end, we used RRBS to identify cell-type-specific demethylation in other tissues, including lung, heart muscle, and hippocampus, and then compared methylation levels in newborn (1 week) and adult (15 weeks) samples. As in the liver, we observed considerable developmentally generated undermethylation in each tissue, but in addition, identified many sites that undergo demethylation postnatally (Fig. 7). In all of these cell types, the DMRs are enriched for H3K4me1 and H3K27ac, but only in their undermethylated state, suggesting that many of these regions represent enhancer-like regulatory elements that get activated during the first few weeks of life (Supplementary Fig. 9). Published RNA-Seq analysis (see Methods) confirmed this idea by demonstrating that many of these tiles are associated with genes that are indeed upregulated postnatally. These findings are consistent with a previous report on demethylation in β-cells^37^.

## Discussion

During development, individual cell types are generated through a serial process of differentiation characterized by programmed changes in gene expression that give each tissue its specific qualities and functions. In mammals, this is accomplished in parallel with alterations in DNA methylation that serve to stabilize the differentiated state^1^. Recent studies have indicated that most of these DMRs correspond to enhancer-like sequences^4,5,38^ that often act as regulatory elements for nearby genes. It has been assumed that these changes take place exclusively during tissue formation in the embryo, yielding mature cell types that then continue functioning with the same stable methylation pattern for the lifetime of the organism. Here we demonstrate that many tissues continue to shape their epigenetic landscape after birth, suggesting that each cell type may undergo post-developmental molding even after the main process of tissue differentiation has terminated.

On the basis of previous studies demonstrating that demethylation is usually carried out through a TET-mediated 5hmC intermediate^39^, we generated a conditional Tet2/Tet3 double mutant that completely inhibited the biochemical removal of methyl groups from their programmed regulatory sites in vivo, thus enabling us to directly test the role of demethylation in the regulation of hepatocyte gene expression. Our studies indicate that in the absence of demethylation, many genes are inhibited in their ability to undergo induced expression postnatally and this is observed to a large extent precisely at those genes that contain DMRs, indicating a direct effect. We have also carried out important control experiments showing that the TET proteins themselves have no influence on target gene transcription once they are already demethylated (Fig. 3). Taken together, these data suggest that demethylation per se is required for generating a proper postnatal gene transcription program. While for some genes this demethylation brings about a relatively large change in RNA levels (Supplementary Fig. 4), its overall role on gene expression in the postnatal liver appears to be more as a fine-tuning mechanism. Nonetheless, these epigenetic events clearly regulate important aspects of liver size and function (Fig. 5).

Previous experiments using reverse epigenetics had already demonstrated that the presence of methyl groups at regulatory sequences serves as an inhibitor of gene expression in vivo^40,41^, but it was not clear how methylation works dynamically in turning on and turning off transcription. It has been demonstrated, for example, that de novo methylation itself does not act as a primary mechanism for repression, but rather takes place after transcription has already been turned off, thus serving as a secondary locking device to prevent gene activation at subsequent stages of development^2,3^. A corollary to this concept is that the removal of methylation must be required to turn on transcription. Support for this idea initially came from studies in differentiated ES cells^42^ and was then proved in vivo by genetically preventing demethylation during B cell-lineage differentiation^24^ and during intestinal stem cell renewal^43^. Our experiments with the Tet2/Tet3 mutants clearly confirm this general principle and lay to rest any doubts^44^ about the role of DNA methylation as an inhibitor of gene expression.

The overall impression from these results is that the liver undergoes programmed postnatal changes in gene expression that are initially mediated by *trans*-acting factors, but then become fixed into the fabric of chromatin much in the same manner that developmental changes in methylation during embryogenesis are retained as an epigenetic memory in the adult. It is very likely that these events occur in response to behavioral or internal environmental changes that take place after birth. We have indeed demonstrated that a significant part of this process is mediated by insulin signaling in the liver. Physiologically, these postnatal alterations are probably triggered by the metabolic transition from a maternal source of energy to the initiation of nursing right after birth and then again by the weaning process 3 weeks later. In keeping with this, many of the demethylation events actually occur in genes involved in energy metabolism (Fig. 2).

The picture that emerges from these studies is that postnatal demethylation can be mediated by hormones and other metabolic signals which affect the expression or activity of key tissue-specific pioneer factors that recognize the gene regulatory regions and facilitate recruitment of the demethylation machinery (Tet2/Tet3). Our experiments (ATAC-Seq) indicate that removal of methyl groups brings about a local increase in accessibility that promotes additional transcription factor-binding in vivo. Indeed, it is likely that while tissue-specific factors may be responsible for priming these regulatory sequences, demethylation could serve to open them further in order to allow the binding of additional transcription factors. In keeping with this hypothesis, an analysis of data obtained from the human ENCODE project indicates that a variety of different general factors bind exclusively to DMRs in each specific cell type, even though there are many other methylated enhancer-like binding sites present in the same cell (Supplementary Fig. 10).

In light of the extensive postnatal methylation changes that take place in different cell types throughout the body, it is tempting to suggest that similar alterations could take place as a result of exposure to environmental influences such as diet, physical activity, pharmacological intervention, disease, or trauma. If this were the case, our results suggest that this might occur through the induction of hormones and other signaling molecules that would then interact with cells, thus signaling a programmed process of changes that brings about a specific pattern of demethylation. Although the trigger for these events might be transient, the concomitant change in DNA methylation may alter expression patterns in a stable manner that can be “remembered” epigenetically even after the original “inducers” are no longer present in these cells^11^.

## Methods

### Animals and cells

C57Bl mice were used for all experiments. DKO mice were generated from Tet2^F^/Tet3^F^ mice^26,45^. A total of 2 × 10^11^ genome copies of AAV8 carrying the gene for Cre under the hepatocyte-specific Tbg promoter (Penn vector) were injected into the superficial temporal vein of 1-day-old mice following anesthetization on ice. Three-week-old mice were anesthetized and injected in the jugular vein with 10^12^ genome copies of this same virus. Liver-specific insulin receptor knockout mice were generated in a similar manner using InsR^F^ mice^34^. All animal experiments were performed in accordance with the guidelines of the Hebrew University Institutional Committee for the Use of Animals for Research.

Mice were anesthetized, sacrificed, and subjected to liver perfusion. Whole liver was then digested by collagenase (Roche) and hepatocytes removed and isolated by means of a percoll gradient^46^. Cells were then stained with a CD-45 antibody (Biotech) and FACS sorted according to size and to negative staining for CD-45. This yielded a population of relatively pure (>95%) hepatocytes. Unsorted liver cells from newborn mice were seeded and grown on collagenase-coated tissue culture plates containing hepatocyte medium supplemented with 5% fetal bovine serum, 1% hepatic growth supplement, 100 U/mL penicillin, and 100 µg/mL streptomycin (ScienCell) for 2–4 weeks.

### RRBS and WGBS

DNA was isolated from mouse hepatocytes or from snap-frozen mouse tissues and incubated in lysis buffer (25 mM Tris-HCl (pH 8), 2 mM ethylenediaminetetraacetic acid, 0.2% sodium dodecyl sulfate, 200 mM NaCl) supplemented with 300 μg/mL proteinase K (Roche) followed by phenol:chloroform extraction and ethanol precipitation and RRBS libraries were prepared^13^ and run on HiSeq 2500 (Illumina) using 100 bp paired-end sequencing. WGBS libraries were prepared using the Ovation^®^ Ultralow Methyl-Seq Library System (NuGEN Technologies Inc.) as per the manufacturer’s instructions. One hundred and fifty nanograms of genomic DNA was sheared with the M220 Focused-ultrasonicator™(Covaris^®^) using the 200 bp snap-cap protocol and subsequently purified with AMPure XP beads. After end repair, adaptor ligation, and final repair, DNA was bisulfite converted with the EpiTect Fast DNA Bisulfite Kit (Qiagen GmbH) following the cycling conditions specified in the Ovation^®^ Ultralow Methyl-Seq Library System protocol. Libraries were then amplified, purified, pooled, and sequenced on the Illumina HiSeq with 70 bp paired-end sequencing. Sequencing was conducted by the University of Pennsylvania Next-Generation Sequencing Core (Philadelphia, PA, USA) at a depth that exceeds the 30-fold “gold standard” commonly achieved in WGBS databases.

### RNA-Seq, ChIP-Seq and 5hmeDIP-Seq

RNA was isolated from mouse hepatocytes using the miRNeasy Kit according to the manufacturer’s instructions (Qiagen). RNA-Seq libraries were generated using the TruSeq RNA Sample Preparation Kit v2 (Illumina). For ChIP analysis, chromatin was fixed and sheared with the M220 Focused-ultrasonicator™ (Covaris^®^) using the microTube snap-cap. Five micrograms of chromatin was then immunoprecipitated with 2 μg of H3K4me1, H3K27ac (Abcam), or HNF6 (Santa Cruz, sc-13050) antibodies. Libraries were generated using the NEBNext Ultra™ II DNA Library Prep Kit for Illumina (New England Biolabs).

The ATAC-Seq protocol^47,48^ was carried out on WT or Tet2/3 DKO hepatocytes with the following modifications. Nuclei were washed with 150 μl of cold phosphate-buffered saline, pelleted at 2000 × *g* at 4 °C for 5 min, re-suspended in 150 μl of cold lysis buffer, and then re-pelleted for 2000 × *g* at 4 °C for 10 min. Nuclei were re-suspended in 150 μl of transposition reaction mix and incubated at 37 °C for 30 min. DNA was isolated (Qiagen MinElute Reaction Cleanup Kit) and subjected to PCR amplification to make the library^48^. Magnetic beads (AMPure XP) were used to purify an ATAC-Seq library^47^, followed by quality control with a high-sensitivity DNA bioanalysis chip (Agilent), quantification with KAPA (Illumina), and QuBit. The libraries were sequenced (75 bp paired-end) on Next-Seq (Illumina).

For 5hMeDIP-Seq, DNA was isolated from hepatocytes harvested from a 20-day-old mice and sheared with the M220 Focused-ultrasonicatorTM (Covaris) using snap-cap tubes. Libraries were generated using the NEBNext Ultra DNA Library Prep Kit (NEB E7645). Library DNA was denatured in 95 °C for 10 min, and then the denatured DNA was precipitated with 5hmC Ab (Active Motif 55010) and sequenced (50 bp single-end) on Next-Seq (Illumina).

### Functional analysis

Blood serum was collected by cardiac puncture from overnight-fasted mice and tested for cholesterol, HDL, and triglyceride levels. Blood glucose levels from overnight-fasted mice were measured by glucose test strips (Roche). Liver tissue was fixed in 4% formaldehyde for 8 h and then preserved in 70% ethanol. Liver was embedded in paraffin, sliced, and mounted on slides, which were then stained with an E-Cadherin antibody and Cherry secondary antibody as well as DAPI (4',6-diamidino-2-phenylindole) (Sigma). Cell size was calculated using the Image J software.

### Data analysis

A chart of all high-throughput experiments is shown in Supplementary Fig. 11. All data have been deposited in the Gene Expression Omnibus (GEO) under accession number GSE85251. DNA methylation was analyzed by using 100 bp paired-end sequencing reads from RRBS that were trimmed and quality filtered by trim galore software (v.0.3.3) using default parameters for RRBS with the following command:

trim_galore–rrbs–paired sample_R1.fastq.gz sample_R2.fastq.gz

Read alignment (genome build mm9) and extraction of single-base resolution methylation levels were carried out by BSMAP v.2.74 (Ref. ^49^) using the following commands:

bsmap -a sample_R1_val_1.fq.gz -b sample_R2_val_2.fq.gz -d mm9_AllGenome.fa -o bam_file -R -D C-CGG

python methratio.py -i “no-action” -p -g -z -o sample_trim_galore_mm9.fa.meth -d mm9_AllGenome.fa bam_file

Percent methylation was calculated for 100 bp tiles with a minimum coverage of 10 CpGs. Hepatocyte-specific undermethylated or de novo methylated regions were at least 50% less or more methylated in five out of nine different tissues. This relatively large cutoff was chosen in order to maximize for specificity and significance. Using these DMRs as our starting material, postnatally demethylated regions were then defined as being at least 35% more methylated (*p* < 0.05) in newborn (1 week) as compared to adult hepatocytes. This slightly lower cutoff was used to take into consideration the possible loss of methylation that takes place immediately after birth, but prior to our first hepatocyte DNA sample (1 week). The same process was performed for tissue-specific demethylation of other tissues (lung, heart, and hippocampus). WGBS was analyzed in a similar manner to the RRBS data. Two hundred base pair DMRs with a minimum coverage of 15 CpGs were identified. In order to find hepatocyte-specific undermethylated tiles at the whole-genome level, we compared our results to a published data set on other tissues (GSE42836). Postnatal tiles were found using similar parameters to those of RRBS.

Genomic feature annotation of DMRs was carried out with annotatePeaks.pl with the promoter defined as −1 kb to + 100 bp of the TSS, intragenic regions including coding exons, the 3′ and 5′ UTRs, introns, and an additional 1 kb beyond the transcription termination sequence. Everything else is considered to be intergenic.

The statistical significance of the original tissue-specific DMR set was calculated using a permutation test: The *p* value was obtained by measuring the quantile of the original DMR size. In each permutation we replaced the same number of samples with random samples from other tissues. Since we had nine tissues and each one of them contained on average six different samples, we could repeat this process numerous times. In the case of hepatocytes, the original number of hepatocyte-specific unmethylated regions was ~9000, and analysis of 10,000 permutations yielded at most five DMRs. The reason for this very low number is that when comparing mixed tissues to other tissues with our very stringent parameters, it yields almost no DMRs, demonstrating the purity of our DMRS. The *p* value was calculated to be smaller than 10^−4^ since for 10,000 permutations almost no DMRs were found. DMRs were also validated by the examination of methylation status on new RRBS and WGBS samples that did not participate in the original analysis. In addition, comparison using methylKit in R with similar cutoff criteria detected 4790 DMRs, of which 93% overlapped with the 9291 DMRs generated by our algorithm (*p* value <10^−100^ as determined by the *χ*^2^ test of homogeneity).

For RNA-Seq, ChIP-Seq and ATAC-Seq sequences were trimmed with cutadapt v.1.15 and aligned with bowtie2 v.2.2.4. Duplicated sequences were removed with samtools rmdup. RNA-Seq 50 bp single-end reads were obtained from HiSeq 2500 and analyzed by TopHat2 (Ref. ^50^). Differential expression was identified by CuffDiff and Cufflink version 2.2.1 (Ref. ^51^) (*p* value ≤0.05). These data were normalized by counting fragments per kilobase million units and dividing by the average of all treatments. Our studies also allowed us to look at the overall effects of preventing postnatal demethylation. RNA-Seq, for example, identified a total of about 870 genes that are expressed at lower levels in the DKO as compared to WT 20-week hepatocytes and many of these (*n*~400) are significantly upregulated postnatally. We also detected 790 genes that are actually upregulated in the knockout as compared to WT. These changes may result from indirect effects downstream of the primary methyl-dependent genes or from activities of the TET proteins not related to DNA methylation^52^. Alternately, they may come about as a compensation response to changes in physiology resulting from the DKO. Expression in non-hepatocyte tissues was derived from published RNA-Seq data (Brain—Adult: ENCFF001LBM, ENCFF001LBR, Newborn: ENCFF037JQC, ENCFF447EXU, ENCFF002EYI, ENCFF002EYD; Lung—Adult: ENCFF001LDK, ENCFF001LDL, Newborn: ENCFF333IFD, ENCFF542DBJ; Heart—Adult: ENCFF001LCD, ENCFF001LCE, Newborn: ENCFF034XQS, ENCFF381RLD).

ChIP-Seq and 5hmDIP-Seq 50 bp single-end reads were obtained from HiSeq 2500 and analyzed by Homer (findPeaks, histone, or factor style) and heatmaps were prepared by Deeptools (computeMatrix). ChIP-Seq distribution was generated by Homer (annotatePeaks.pl). The Genome browser view was generated by converting Bam files into using the bedGraphToBigWig tool and then uploaded on the IGVtools (count). ChIP-Seq data were obtained from the publicly available GEO database, including H3K4me1 and H3K27ac data from the liver, lung, heart, and hippocampus collected from the ENCODE website (https://www.encodeproject.org/), HNF4α (GSM1390711), RXRa (GSM864674), PPARα (GSM864671), and DNaseI (GSM542592). Motif analysis was carried out by HOMER (findMotifsGenome.pl). For ATAC-Seq 75 PE sequences were aligned and duplicated as well as mitochondrial DNA was removed before peak calling by homer.

Published liver Hi-C datasets (GSE65126) were aligned to the mouse genome (mm9) using Bowtie. Aligned reads were analyzed using HOMER, filtering out paired reads that map within 1 kb of each other that map to regions with at least 5× higher than average sequencing coverage or that are not within 500 bp of a *Hin*dII site. Interactions with a *p* value <0.01 and a modified *z*-score >1.5 were calculated with the analyze Hi-C interactions tool in the HOMER package, using a background model of 10 kb bins. Interactions between promoters and DMRs were identified from this list using the HOMER annotateInteractions.pl tool. Genes that show down-regulation in the Tet2/3 DKO were identified from the above list.

### Data availability

The sequencing data have been deposited in the GEO under accession number GSE85251. Accession codes for previously available datasets also used here are listed in the data analysis section. The data that support the findings of this study are available from the corresponding author upon reasonable request.

## Electronic supplementary material

Supplementary Information

Description of Additional Supplementary Files

Supplementary Data 1
