# Supplementary material for: Postnatal DNA demethylation and its role in tissue maturation
Source: Nat Commun. 2018 May 23;9:2040. doi: 10.1038/s41467-018-04456-6 (PMC5966414; doi:10.1038/s41467-018-04456-6)
Supplement: Supplementary file 1 — Supplementary Information [file 41467_2018_4456_MOESM1_ESM.pdf]

Postnatal DNA demethylation and its role in tissue maturation

Reizel et al.

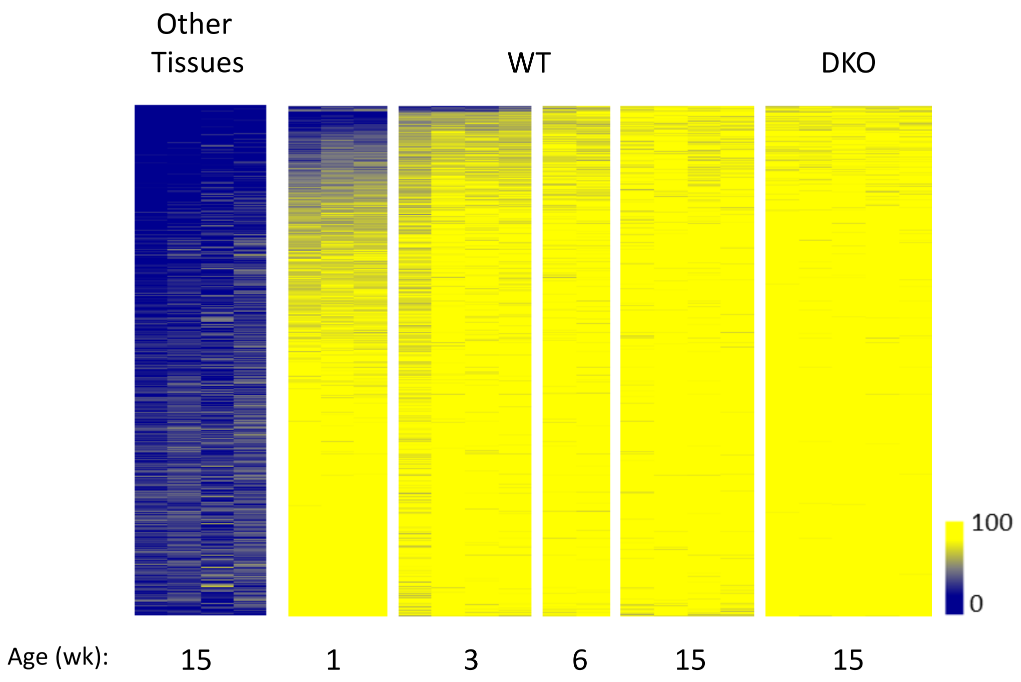

Supplementary Fig. 1. Tissue-specific de-novo methylation in hepatocytes. Heatmap of hepatocyte-specific de novo methylated tiles (n~6,000, RRBS) in multiple samples (n=2-7) from WT and DKO hepatocytes at different times postnatally. Also included are samples from other adult tissues (heart, neutrophils, brain, fat). Yellow represents high and blue represents low DNA methylation levels. Only ~400 tiles (<10%) undergo de novo methylation postnatally as opposed to during embryonic development.

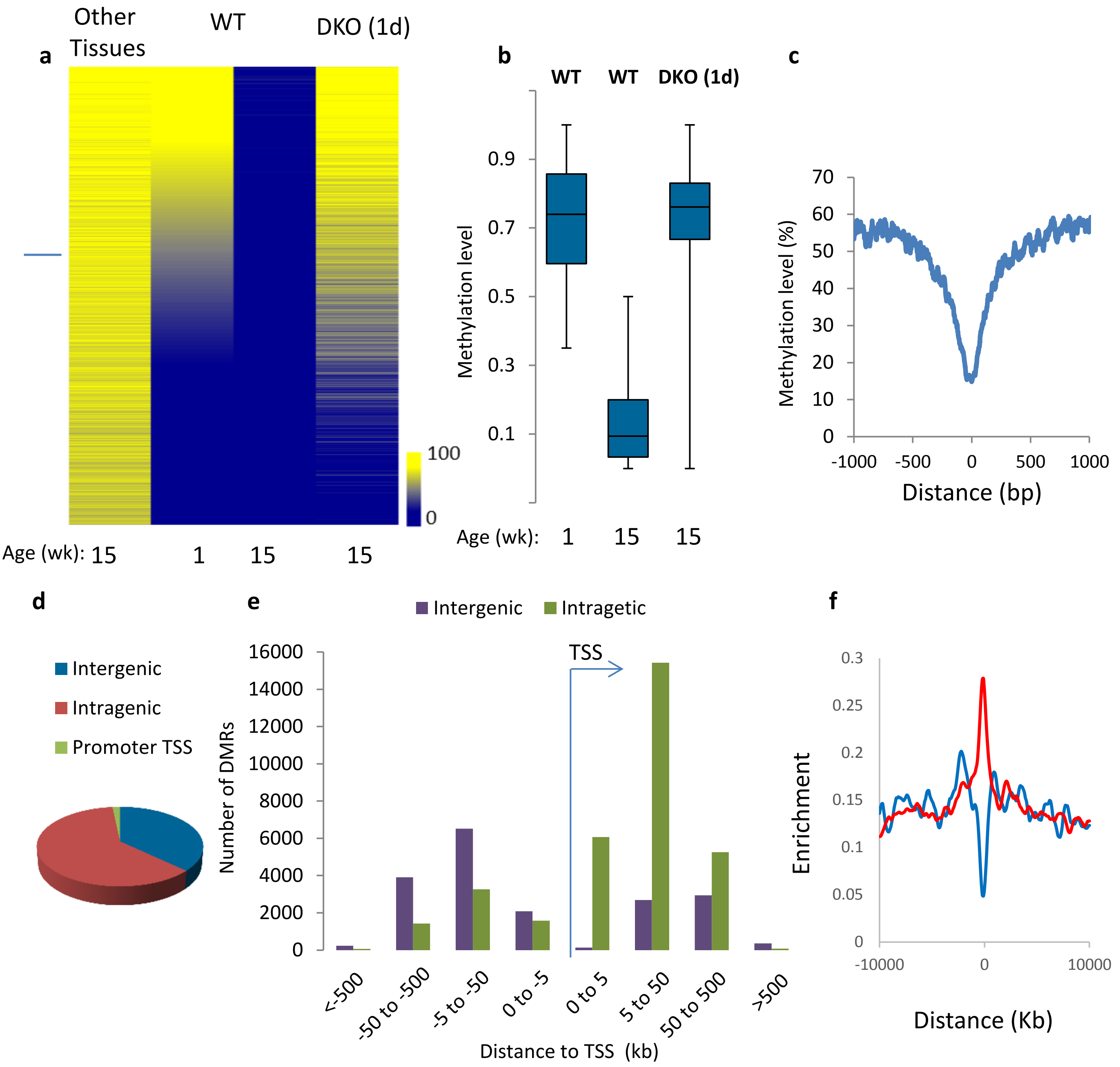

Supplementary Fig. 2. Postnatal hepatocyte demethylation.
a Methylation (WGBS) heatmap of hepatocyte-specific tiles (n~120,000) in newborn (1wk) or adult (15wk) WT and DKO mice, ranked according to 1wk hepatocytes and compared to DNA from other tissues. Each column represents a mixture of four individual biological replicates. The blue line indicates the cutoff for postnatal (n=52,000) as opposed to prenatal (n=70,000) DMRs. The DKO was carried out by AAV8 injection in 1d newborns. Yellow represents high and blue represents low DNA methylation levels. **b** Box plot of DNA methylation levels for postnatal DMRs in WT and DKO hepatocytes. **c** Distribution of DNA methylation levels at each CpG (WGBS) for all DMRs in the liver as a function of distance from the original 100-bp tiles detected by RRBS. 96% of the DMRs are < 400bp in length. **d** Genomic distribution of postnatal DMRs. There is a 2.5 fold enrichment of DMRs in intragenic regions (p<0.01). **e** Number of intra- or intergenic DMRs as a function of distance from the nearest TSS in the genome (annotatePeak).
f Distribution of 5hmC in WT hepatoctyes (3wk) as a function of distance from the center of each prenatal (blue) or postnatal (red) DMR (RRBS, n=2,800).

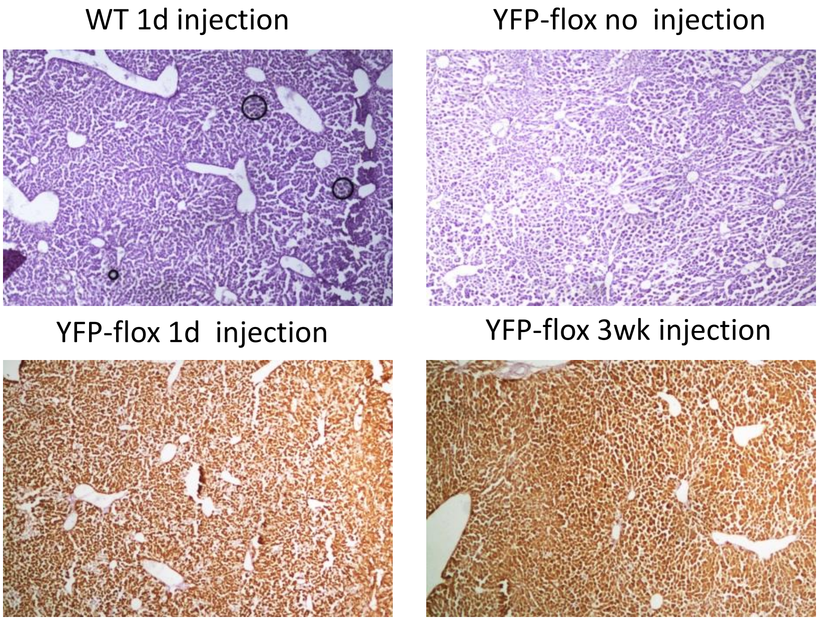

Supplementary Fig. 3. Tet2/3 DKO validation.
As an indication for Tet2/3 deletion and in order to titer viral efficiency, mice carrying a YFP hepatocyte reporter with a flox-flanked stop codon (YFP-flox) [1] and WT mice were injected with AAV8-Cre at either 1wk or 3wk and then assayed for YFP by immunohistochemistry on liver sections about 1wk after the injection. This typically showed >97% knockout efficiency. Quantitative PCR analysis of DNA from 20wk DKO hepatocytes confirmed that deletion of both genes was almost complete (>95%).

DMRs	Intragenic		Intergenic	Total
Gene induction	Yes	No		
Number	8344	22659	21023	52026
Δmethylation	61	59	60	60
H3K4me1 (%)	94	90	85	89
H3K27ac (%)	85	70	59	68
DNase I (%)	33	27	31	30
ATAC (%)	16	23	21	21
PPARα (%)	72	65	65	66
RXR (%)	48	47	55	50
HNF4α (%)	44	45	43	44
HiC interactions with postnatally induced genes (n=2077)	506	748	692	1189
HiC interactions with genes inhibited in the DKO (n=390)	108	129	158	237

Supplementary Fig. 4. Characterization of DMR chromatin structure.
DMRs were divided into three categories: 1) within gene regions that undergo postnatal induction, 2) within gene regions that do not undergo postnatal induction and 3) intergenic regions and then assayed for histone modification patterns (H3K4me1, H3K27ac), accessibility (DNaseI, ATAC) or transcription-factor binding (PPARα, RXR, HNF4α) as a percent of DMRs in that category detected be the peak finder. The number of induced-gene interactions or the number of DKO-inhibited gene interactions in each category as determined by HiC is also shown (lower panels). The last column represents all of the unique interacting genes after subtraction of overlap within the three categories. Note that these interactions can explain approximately 60% of the methylation effects (1189/2077=57%, 237/390=61%). Over 30% of the DMRs within induced genes interact with the host gene, while 0% of the DMRs in the non-induced genes interact with the host gene.

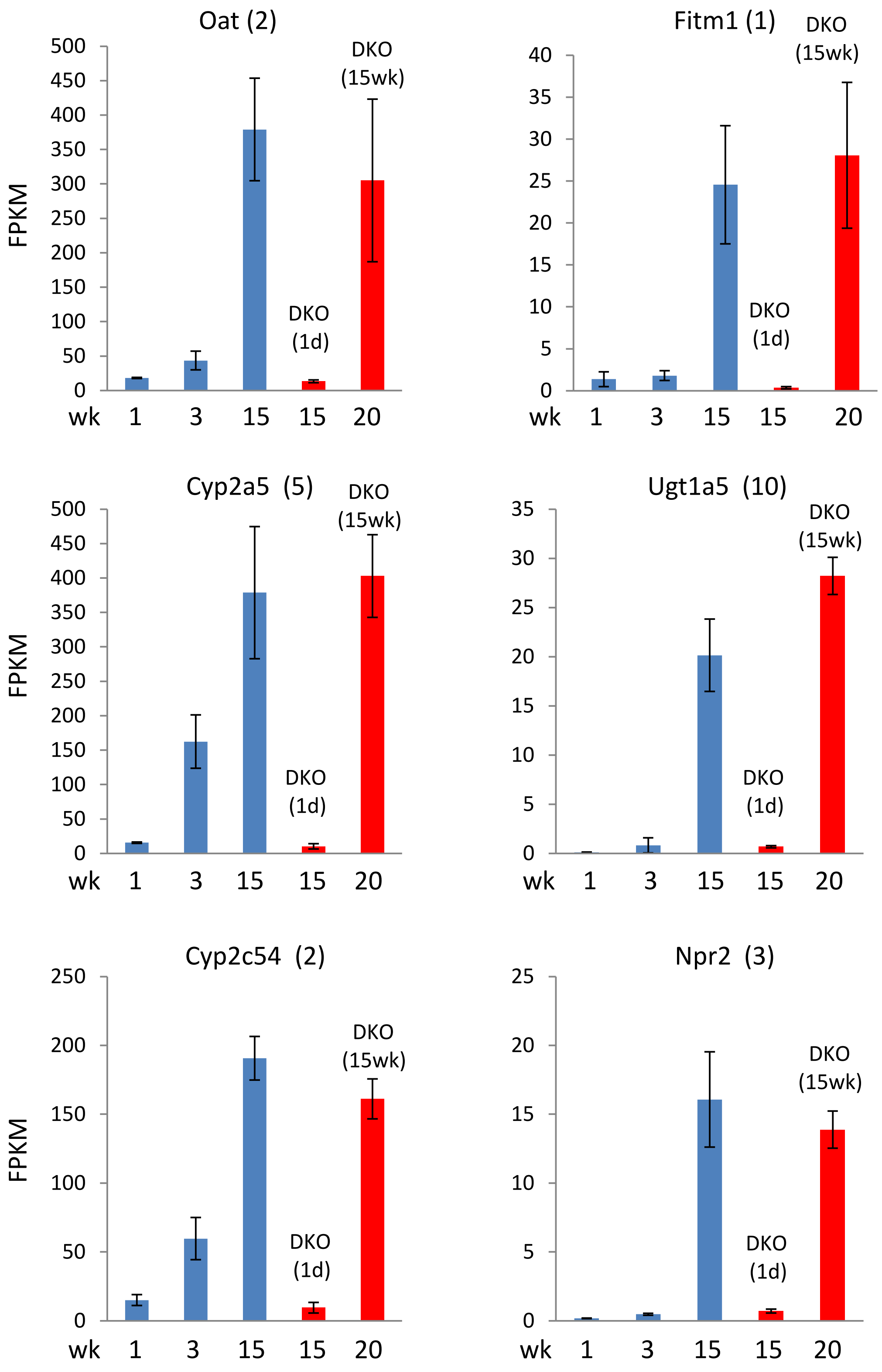

Supplementary Fig. 5. Demethylation and gene expression.
Specific examples of WT (blue) hepatocyte RNA expression levels for selected genes associated with postnatal DMRs (WGBS) as a function of time after birth and for the Tet2/Tet3 deletion (red) carried out at either 1d or 15wk. Note that in all these cases the early deletion prevents gene induction ($P<0.001$ for all examples), while deletion at 15wk (after demethylation has already taken place) has no effect on expression. The number of DMRs associated with each gene is shown in parenthesis.

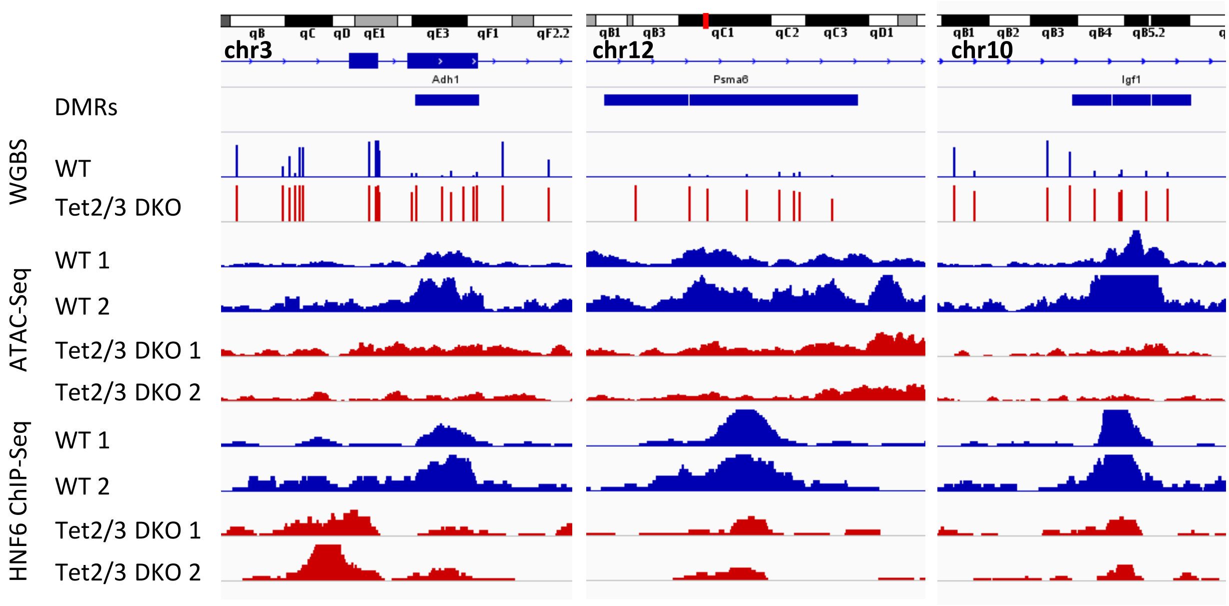

Supplementary Fig. 6. Effect of Tet2/Tet3-DKO on local chromatin structure. Two biological replicates from normal or DKO (1d) 15wk hepatocytes were subjected to ATAC-Seq and HNF6 ChIP-Seq and the results, together with WGBS, mapped on three specific gene regions. The DMRs are marked in blue. All of these genes are induced postnatally in WT, but not DKO mice.

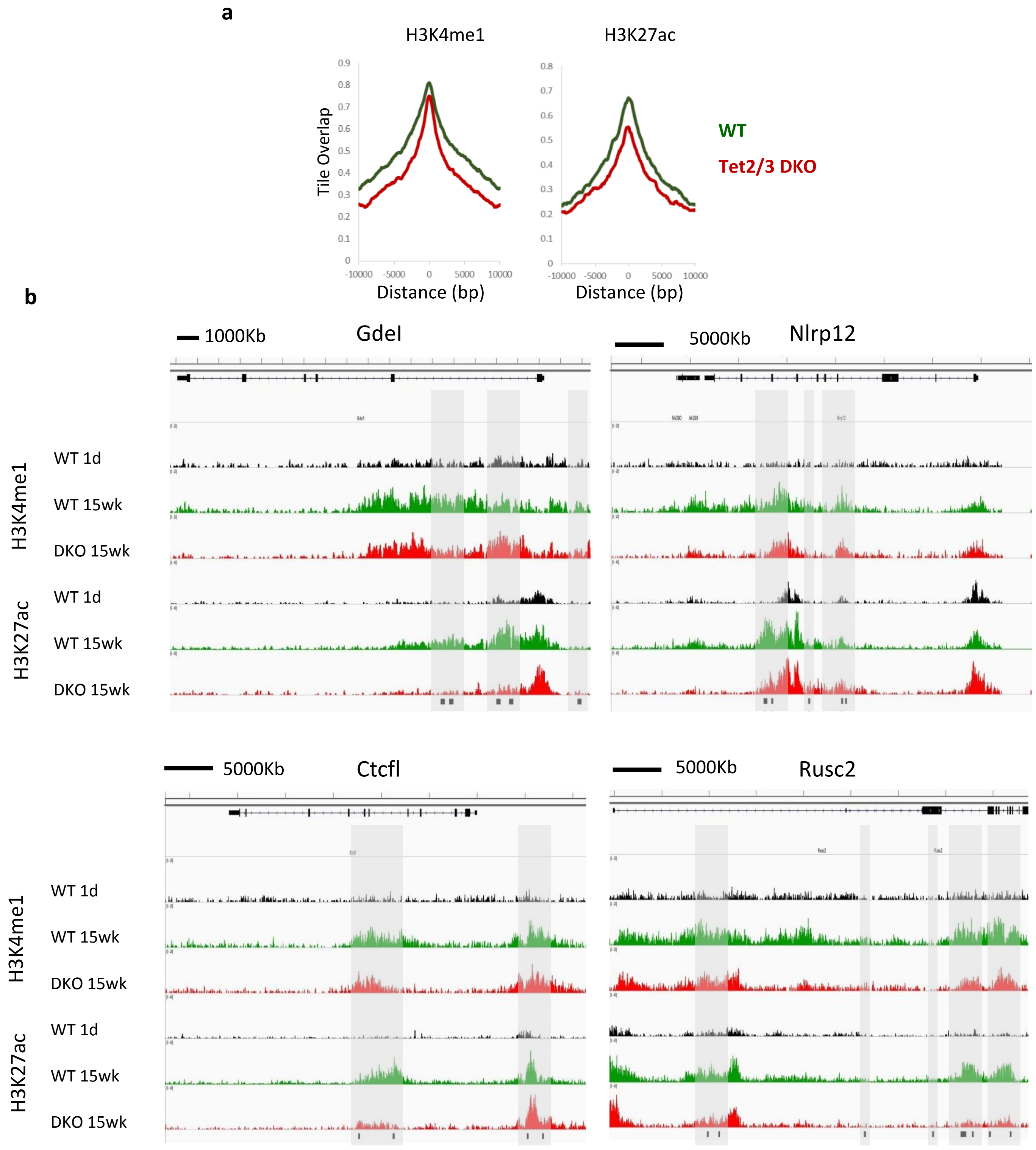

Supplementary Fig. 7. Histone modification at DMRs.
a Quantitative H3K4me1 and H3K27ac ChIP-Seq of DMRs measured in 15wk WT (green) and DKO (red) hepatocytes as a function of distance from their center tile. Only DMRs associated with genes whose expression was affected by the DKO were selected (n=1,995).
b Browser display of quantitative H3K4me1 and H3K27ac on selected genes. Data for newborn liver was derived from the ENCODE website. The location of all DMRs are highlighted and marked in black.

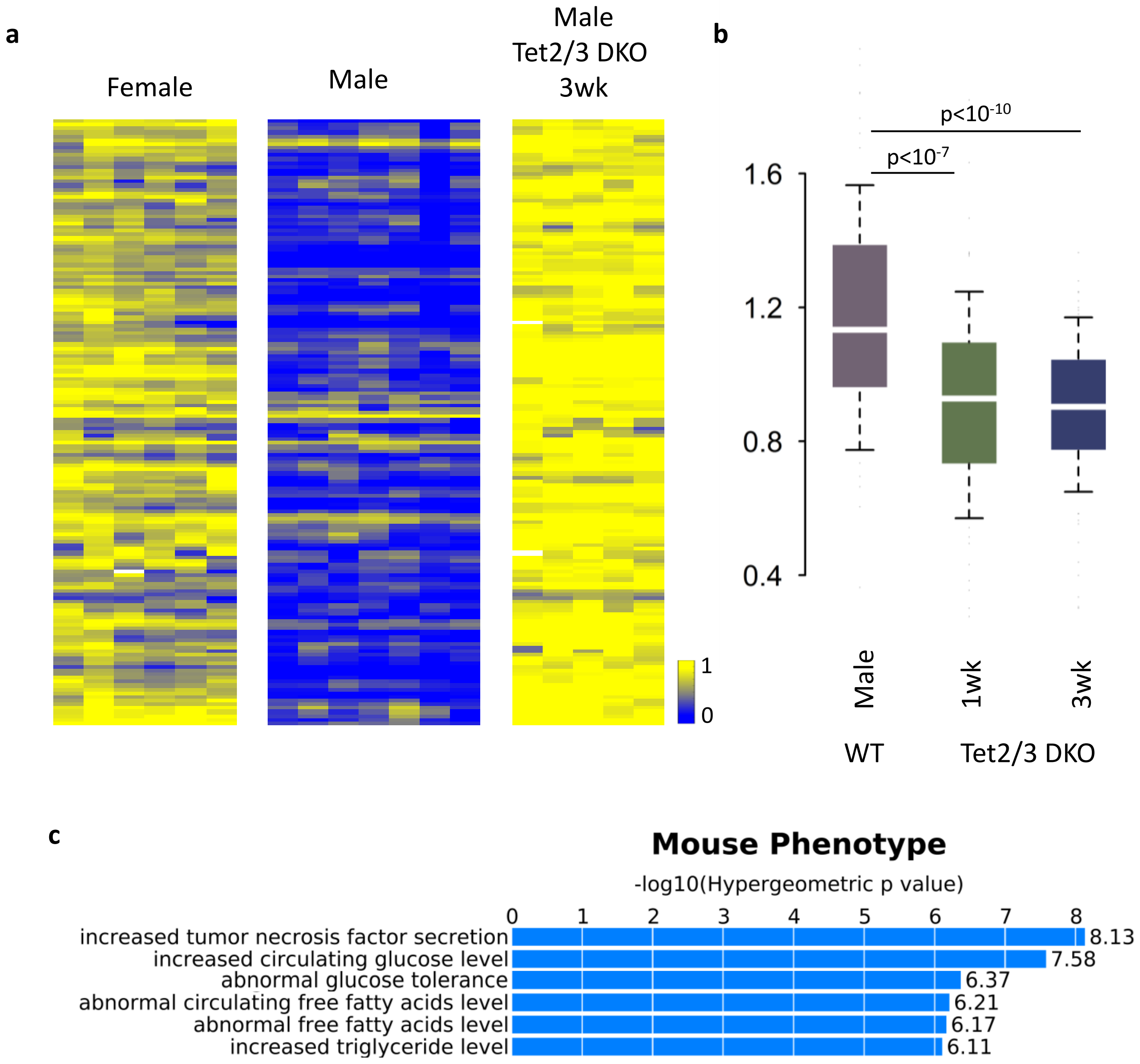

Supplementary Fig. 8. Male/female differential DMRs.

a Heatmap of 160 DMRs[2] that differ between male and female in 15wk hepatocytes showing normal and Tet2/Tet3-DKO male mice.

b RNA-Seq analysis of 88 genes associated with male-specific DMRs in normal and DKO hepatocytes (measured at 15wk).

c GO analysis of genes associated with male-specific DMRs.

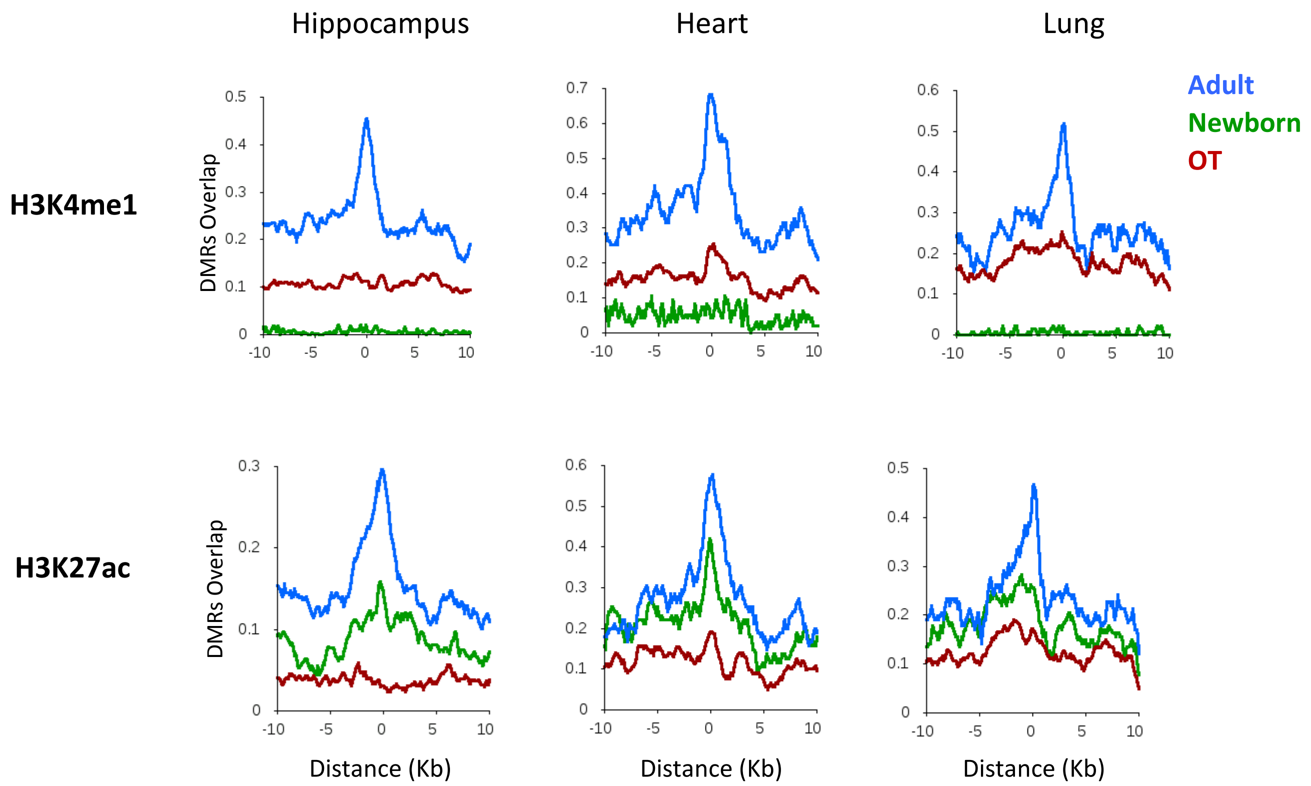

Supplementary Fig. 9. DMR histone modification in other tissues.
ChIP-Seq of tissue-specific DMRs (RRBS) as a function of distance from their center tile for H3K4me1 and H3K27ac in the relevant tissue or in a mixture of other tissues (OT) for heart, lung and hippocampus.

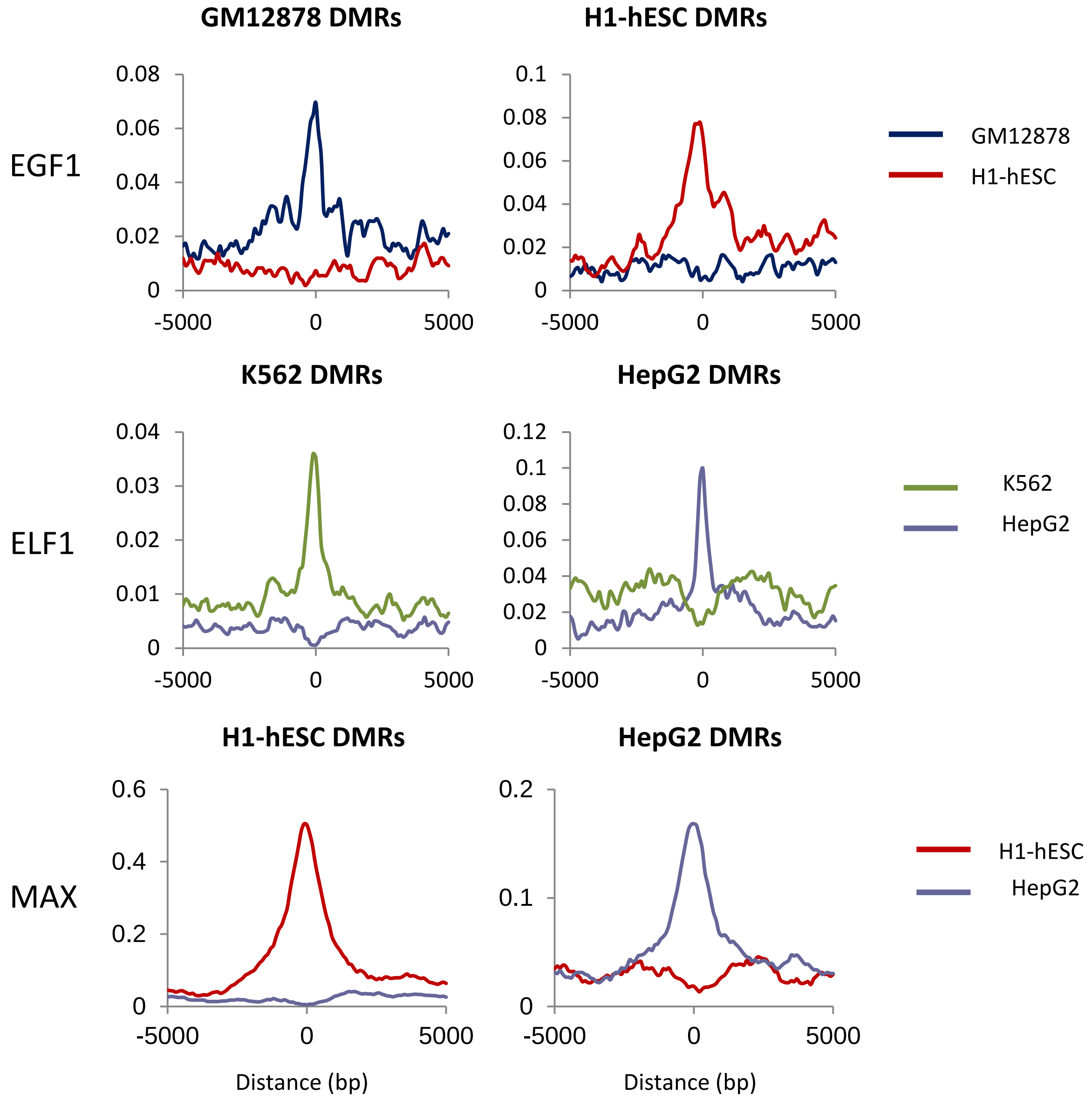

.Supplementary Fig. 10. Factor binding vs. DNA methylation
DMRs for each of four different human cell lines, GM12878 (lymphoblast), H1-hESC (embryonic stem cells), K562 (erythroleukemia) and HepG2 (liver) by identifying tiles that are >50% less methylated (RRBS) as compared to the other three cell types. ChIP-Seq data for three different transcription factors (EGF1, ELF1, MAX) was used to determine the binding distribution of these factors around the DMRs (distance in bp) in each cell type. These data indicate that any given factor binds to the undermethylated regions (DMRs) in each cell type, while the same factor does not bind at these sites in another cell type where they are methylated. Thus, for example, MAX binds to the H1-hESC specific DMRs in H1-hESC, but not to these same tiles in HepG2 cells, even though the factor is present in both cell types as shown by the observation .that MAX does bind the DMR set specific to HepG2 cells

All data was obtained from the human ENCODE project. Percent methylation was calculated for two-hundred-base pair tiles with a minimum coverage of 10 CpGs. Tissue-specific undermethylated regions were at least 50% less methylated in 2 out of 2 or 3 out of 4 different tissues. ChIP-Seq peaks for each factor in each cell line were generated by Homer .((annotatePeaks.pl

Treatment	mice strain	Age (wk)	Methods						
			RRBS	WGBS	RNA-Seq	ATAC-Seq	Chip-Seq (Histone)	ChiP-Seq (Factor)	5hMeDIP-Seq
WT	C57bl	1	3	pool (3)	2				2
		3	4		3				2
		6	2						
		12	4						
		15	4	pool (3)	3	2	2	2	
		20	3						
CRE virus injection	Control 1d	15	4		3				
	DKO 1d	15	5	pool (3)	3	2	2	2	
	Control 3wk	15	4		3				
	DKO 3wk	15	5		3				
	DKO 15wk	20	3		4				
	in vitro	1	7						

Supplementary Fig. 11. Chart of high throughput analysis.
The samples analyzed by high throughput techniques are shown together with the number of biological replicates included in each.

Supplementary References

1. Srinivas, S. et al. Cre reporter strains produced by targeted insertion of EYFP and ECFP into the ROSA26 locus. *BMC Dev. Biol.* **1**, 4 (2001).

2. Reizel, Y. et al. Gender-specific postnatal demethylation and establishment of epigenetic memory. *Genes Dev.* **29**, 923-933 (2015).
