# Supplementary material for: Postnatal DNA demethylation and its role in tissue maturation
Source: Nat Commun. 2018 May 23;9:2040. doi: 10.1038/s41467-018-04456-6 (PMC5966414; doi:10.1038/s41467-018-04456-6)
Supplement: Supplementary file 2 — Description of Additional Supplementary Files [file 41467_2018_4456_MOESM2_ESM.pdf]

Descriptions of Additional Supplementary Files

File Name: Supplementary Dataset 1
Description: 52,000 DMRs
